# Effect of Climatic Changes

**Published:** 1891-03

**Authors:** 


### EFFECT OF CLIMATIC CHANGES.

The changes of the seasons are wonderfully advantageous. In the summer, Nature slows down all the vital fires. It is necessary to run the vital machinery slower, in order to preserve life. We become more sluggish, and lose our energy and ambition. This is noticeable in all warm countries. It takes a larger force of men a greater length of time to do a piece of work in the south than it does in the north, and if a northern man goes south and attempts to work as he formerly did, he will either die or learn to submit to the inevitable laziness of the climate. The people of warm countries never put on a winter constitution, and naturally fall into this slow summer rut.

Here in the north, we get the tonic of cold weather. We breath a little more rapidly ; we think faster ; we are more energetic ; we are lifted to a higher plane of existence. Thus many of those who run away to the south at the first approach of cold weather, suffer great loss. It is well enough for those who are so weakened that they cannot put on a winter constitution; but to those who are able to tone themselves up to freedom of out of door exercise, it is the greatest possible blessing to remain in the more invigorating climate of the north. The most energetic peoples in the world are those who live in the temperate zones, and are inured to climate changes. They embrace also the largest and the finest specimens of humanity.

